# Identifying Risk Factors for Caregiver Burden in Neurological Disorders Using Machine Learning

**DOI:** 10.3390/medsci14040428

**Published:** 2026-07-25

**Authors:** Maria Grazia Maggio, Augusto Ielo, Rosaria De Luca, Francesco Corallo, Angela Marra, Davide Cardile, Amelia Rizzo, Angelo Quartarone, Rocco Salvatore Calabrò

**Affiliations:** 1 IRCCS Centro Neurolesi Bonino-Pulejo, Strada Statale 113 - C.da Casazza, 98124 Messina, Italy; mariagrazia.maggio@irccsme.it (M.G.M.); rosaria.deluca@irccsme.it (R.D.L.); francesco.corallo@irccsme.it (F.C.); angela.marra@irccsme.it (A.M.); davide.cardile@irccsme.it (D.C.); angelo.quartarone@irccsme.it (A.Q.); 2 Department of Clinical and Experimental Medicine, University of Messina, Piazza Pugliatti, 1, 98120 Messina, Italy; amrizzo@unime.it

**Keywords:** caregiver burden, stroke, Parkinson’s disease, Alzheimer’s disease, cognitive impairment, machine learning, dyadic factors

## Abstract

**Background:** Caregiver burden represents a multidimensional syndrome influenced by patient-related, relational, and contextual factors in neurological disorders. Although stroke, Parkinson’s disease (PD), and Alzheimer’s disease (AD) differ in clinical trajectory, comparative analyses of caregiver risk profiles across these conditions remain limited. **Objective:** This study aimed to identify sociodemographic, cognitive, and dyadic factors associated with caregiver burden in a clinical cohort, and to investigate their value for caregiver risk stratification using supervised machine learning models. **Methods:** In this monocentric observational cohort study, 113 patient–caregiver dyads (79 stroke, 19 PD, 15 AD) were consecutively enrolled in a neurorehabilitation setting. Patients underwent cognitive assessment with the Montreal Cognitive Assessment (MoCA), while caregivers completed the Caregiver Burden Inventory (CBI), which was considered the primary outcome measure. Caregiver burden was dichotomized into mild versus moderate-to-severe burden using established CBI cutoff thresholds. Group comparisons, correlation analyses (false discovery rate–corrected), and supervised machine learning models (logistic regression, random forest, AdaBoost, support-vector machine, naïve Bayes, and CatBoost) were performed using 5-fold stratified cross-validation repeated 10 times. **Results:** Disease-specific burden patterns emerged. Stroke caregivers reported higher time-dependent burden, whereas AD caregivers showed greater emotional burden (*p* < 0.05). No significant differences were observed in total CBI scores across groups. Lower MoCA scores were moderately associated with higher total and time-dependent burden (r up to −0.49, *p* < 0.001), particularly when interacting with advanced patient age. Machine learning models showed moderate performance, with AdaBoost achieving the highest accuracy (75.8%) and logistic regression and CatBoost the highest area under the receiver-operating-characteristic curve (AUC = 0.80). The most influential predictors were the age × MoCA interaction, MoCA score alone, and patient–caregiver gender concordance. Cognitive impairment, especially in older patients, and dyadic gender concordance emerged as central risk factors. **Conclusions:** Multidimensional assessment and early risk stratification, potentially supported by machine learning tools, may improve identification of vulnerable caregivers and guide tailored interventions.

## 1. Introduction

Caregiver burden represents a multidimensional and clinically relevant construct, encompassing the physical, psychological, and social strain experienced by individuals providing care to patients with chronic conditions. In neurological disorders, this burden is often amplified due to the progressive or disabling nature of these conditions, which affect patients’ cognitive, motor, and behavioral functioning. Among the most common and disabling neurological conditions are stroke, Parkinson’s disease (PD), and Alzheimer’s disease (AD). Stroke is an acute cerebrovascular event that causes focal neurological deficits, which can lead to long-term motor, cognitive, and language impairment [1].

PD is a progressive neurodegenerative disease that primarily affects the dopaminergic system, causing bradykinesia, rigidity, tremor, and a range of non-motor symptoms [2].

AD is the most common cause of dementia, characterized by gradual cognitive decline, memory loss, and behavioral disturbances [3].

Although they differ in etiology and progression (being stroke an acquired disease), these conditions share a profound impact on patients’ motor, cognitive, and functional abilities. According to recent data from the Global Burden of Disease Study 2019, the prevalence of neurological disorders in Italy has increased by 13%, demonstrating their growing public health importance [4].

The progressive and disabling nature of many neurodegenerative diseases, such as PD and AD, and the often sudden but long-lasting consequences of stroke, profoundly affect not only patients but also their caregivers [5,6,7].

The Italian National Institute of Health defines a caregiver as anyone who provides care to a dependent person, distinguishing between paid professionals and informal caregivers, typically family members [8]. In this context, caregiver burden is recognized as a complex clinical syndrome, the intensity of which is influenced by several interconnected factors [9,10].

Among the most identified risk factors are the patient’s clinical status, particularly the presence of neuropsychiatric symptoms, and premorbid personality traits [11]. In PD, motor fluctuations and non-motor symptoms, such as depression or apathy, can significantly increase perceived burden. In AD, cognitive decline and behavioral disturbances are often the most challenging. Finally, in stroke, residual physical disability and cognitive impairment may require ongoing care. Caregiver sociodemographic characteristics also play a fundamental role [12].

For clarity, the main factors associated with caregiver burden are summarized in Table 1.

For example, adherence to gender role stereotypes can increase perceived burden and reduce the likelihood of seeking support. Although these dynamics are more closely linked to gender roles than biological sex, studies have shown that women often report higher levels of burden due to social expectations, while men with strong adherence to traditional ideals of masculinity tend to avoid seeking help [13].

The experience of caregiving also varies depending on the relationship between caregiver and patient, with spouses and children exhibiting different levels of burden, identity shifts, and social isolation [14]. Additional predictors of high burden include a lack of social support and the presence of anxiety or depression in caregivers [15]. In conclusion, a caregiving burden is a syndrome that reflects the physical energy and time required to care for a loved one with a neurological condition. It often results in a loss of personal time and space, a redefinition of the caregiver’s role, and emotional distress related to the patient’s illness [9]. In response to this problem, psychoeducational interventions and family-centered approaches have been developed to improve caregivers’ quality of life and promote collaboration between caregivers and healthcare providers [16].

Despite these initiatives, understanding the most effective strategies for addressing caregiver burdens remains insufficiently explored, based on individual caregiver profiles and specific care settings. Identifying caregivers’ specific risk factors and unmet needs is therefore crucial for guiding preventive and supportive interventions, as these factors are consistently highlighted in the existing literature [17,18]. This gap limits the ability to identify targeted and personalized strategies to support caregivers across different neurological conditions.

Alongside conventional regression-based approaches, machine learning methods are increasingly being used in caregiver research to model multidimensional outcomes such as burden, quality of life, and psychological distress [19]. Recent studies have applied machine learning techniques to predict caregiver burden in amyotrophic lateral sclerosis [20] and caregiver quality of life in the same population [21]. Other recent caregiving studies have also explored predictive modelling approaches for psychological distress, particularly among informal caregivers of people with dementia [22]. However, these applications are often disease-specific. In this context, a comparative and integrative approach is needed to better understand both shared and condition-specific determinants of caregiver burden across neurological conditions. To address this gap, we combined conventional inferential statistical analyses to identify factors associated with caregiver burden with complementary machine learning models to explore their potential for caregiver risk stratification. The primary objective of this study is to explore risk factors associated with caregiver burden in a clinical sample of patients with neurological disorders and focus specifically on PD, AD, and stroke, their caregivers. We aimed to identify the sociodemographic, clinical, cognitive, and relational factors associated with higher caregiver burden and to explore whether their combined information could support classification of caregivers at greater burnout risk.

## 2. Materials and Methods

### 2.1. Study Population

This single-centre observational cross-sectional study was conducted at the IRCCS Centro Neurolesi “Bonino-Pulejo” (Messina, Italy). Patient–caregiver dyads were consecutively screened in the Neurorehabilitation Units between November 2022 and January 2026. During the study period, all patients with a diagnosis of stroke, PD, or AD were evaluated for eligibility. Inclusion criteria were: (i) confirmed neurological diagnosis; (ii) presence of a primary informal caregiver providing regular assistance; (iii) clinical stability allowing neuropsychological assessment; and (iv) written informed consent from both patient and caregiver. Dyads were excluded if they did not meet inclusion criteria or declined participation.

### 2.2. Ethics

This study was conducted within a broader rehabilitation research protocol approved by the Local Ethics Committee of IRCCS Centro Neurolesi “Bonino Pulejo” (protocol code 47/21, approved on 6 October 2022) and specifically examines caregiver-related outcomes. Written informed consent was obtained from all participants prior to enrollment.

### 2.3. Procedures

Following eligibility screening and written informed consent, patient–caregiver dyads underwent a standardized assessment protocol conducted within the neurorehabilitation setting. All evaluations were performed by trained neuropsychologists under uniform conditions.

Patients completed a cognitive assessment during a single session. Caregivers completed self-report measures independently, with supervision available if clarification was required. Sociodemographic and clinical information were collected from medical records and structured interviews.

### 2.4. Outcome Measures

Patients’ global cognitive performance was assessed using the Montreal Cognitive Assessment (MoCA), a clinician-administered standardized screening tool evaluating attention, executive functions, memory, language, visuospatial abilities, abstraction, and orientation. Total scores range from 0 to 30, with higher scores indicating better cognitive functioning. Caregiver burden was measured using the Caregiver Burden Inventory (CBI), a multidimensional self-report questionnaire assessing five domains: time-dependence, developmental, physical, emotional, and social burden. The total score reflects overall caregiving strain, with higher scores indicating greater perceived burden.

Caregiver burden was assessed at a single time point within a cross-sectional study design; therefore, no follow-up period was available.

To classify caregiver burden, established CBI cutoff thresholds previously adopted in the caregiving literature were applied. Specifically, a total CBI score ≥ 36 was considered indicative of a high risk of caregiver burnout, whereas scores between 25 and 36 indicated clinically relevant caregiver burden requiring respite or structured support. Scores ≤ 24 were classified as absent or mild caregiver burden [23,24].

### 2.5. Statistical Analysis

To characterize the cohort and evaluate potential differences across pathology groups, the clinical dataset was analyzed with descriptive and inferential statistics. Statistical analyses were conducted in Python 3.11, using libraries including Pandas, NumPy, SciPy and Scikit-posthoc and Pandas. Categorical variables including patient sex, caregiver sex, the caregiver-patient relationship, and caregiver-burnout risk were expressed as counts and percentages for the whole cohort and for each pathology group (stroke, PD, AD). Continuous variables including patient age, MoCA score, years of education, caregiver age, the CBI sub-scores and total score were reported as mean ± standard deviation (SD). For categorical variables, $\chi^{2}$ tests of independence were used to compare distributions across pathology groups. For continuous variables, the Shapiro–Wilk test assessed normality within each group and Levene’s test assessed homogeneity of variance. When both assumptions were satisfied, differences among the three pathologies were examined with one-way analysis of variance (ANOVA); a significant F test was followed by Tukey’s honest significant-difference comparisons. When at least one assumption was violated, the non-parametric Kruskal-Wallis H test replaced ANOVA, and significant results were explored with pair-wise Dunn tests. All post-hoc *p*-values were adjusted for multiple comparisons by the Benjamini-Hochberg false-discovery-rate method.

Pair-wise correlations among numeric predictors and caregiver-burden outcomes were then explored. The normality of each variable was assessed with the Shapiro-Wilk test. Pearson correlations were computed when both variables were normally distributed, otherwise Spearman correlations were used. P-values were adjusted for multiple testing with the Benjamini-Hochberg false-discovery-rate procedure. To focus on associations of at least moderate magnitude, only correlations with an absolute coefficient |r| ≥ 0.30 were retained for interpretation. Correlation analyses were run on the full set of candidate predictors, which comprised the original clinical variables and the composite features described in the machine learning framework section.

### 2.6. Machine Learning Framework

In addition to conventional inferential analyses aimed at identifying factors associated with caregiver burden, supervised machine learning models were applied as a complementary exploratory approach to evaluate whether the combination of clinical and dyadic variables could support caregiver risk stratification.

All machine learning analyses were run in Python 3.11 using scikit-learn and CatBoost libraries. The dependent variable was caregiver burden, dichotomized a priori into mild (original class 1) versus moderate-to-severe (original classes 2–3) according to established CBI cutoff thresholds.

#### 2.6.1. Feature Selection and Feature Engineering

Eight clinical descriptors (patient pathology as a categorical variable, patient sex, patient age, years of education, MoCA score, caregiver-patient familiarity (defined as the type of relationship between the caregiver and the patient, e.g., spouse, child, or other), caregiver sex and caregiver age) were complemented with eleven composite features designed to capture age-, sex- and familiarity-related interactions (e.g., absolute age difference, age-to-MoCA interaction, same-gender indicator). The full list of variables is reported in Supplementary Table S1.

#### 2.6.2. Machine Learning Models

Six supervised learning methods were examined: logistic regression, random forest, AdaBoost, support-vector machine (SVM) with a radial-basis-function (RBF) kernel, naïve Bayes and CatBoost. These models were selected to represent different modeling families, including linear, probabilistic, kernel-based, and tree-based ensemble approaches, in order to explore whether caregiver risk stratification patterns were robust across algorithms with different assumptions and different capacities to capture non-linear relationships and feature interactions. For each algorithm, hyperparameters were fixed in the main analysis based on the best-performing configurations identified in a preliminary grid-search procedure. These settings were then kept constant throughout the repeated cross-validation process to avoid repeated tuning during performance estimation. The final parameter sets are reported in Supplementary Table S2.

For models that allow weighting of observations, automatic class weighting was enabled to counterbalance the class imbalance between caregivers at mild and moderate/severe risk of burnout. No synthetic oversampling or bootstrap resampling was applied. Random seeds were fixed to ensure reproducibility.

#### 2.6.3. Cross-Validation Protocol

Internal validation performance was estimated using 5-fold stratified cross-validation repeated ten times with different splits (50 train/test iterations in total). Repeated cross-validation was selected to make efficient use of the limited sample and to reduce dependence on a single train–test partition. At each iteration, models were trained on the training folds and evaluated on the corresponding held-out fold, while preserving the original class distribution. For all models except CatBoost, categorical predictors were encoded within a pipeline-based preprocessing procedure fitted on the training data and then applied to the test fold, in order to prevent data leakage. CatBoost was instead fitted using its native handling of categorical variables.

#### 2.6.4. Evaluation Metrics

Model performance was evaluated in terms of classification and discriminative ability for caregiver burnout-risk stratification. For every fold and algorithm, the following metrics were recorded on the held-out test fold: accuracy, precision, recall, F1-score (positive class = moderate-to-severe), and area under the receiver-operating-characteristic curve (AUC). Accuracy reflects the overall proportion of correctly classified caregivers; precision indicates the proportion of caregivers predicted as moderate-to-severe who were correctly classified; recall indicates the proportion of true moderate-to-severe cases correctly identified; F1-score summarizes the balance between precision and recall; and AUC quantifies the model’s discriminative ability across probability thresholds, i.e., its ability to rank caregivers with moderate-to-severe risk higher than those with mild risk. Mean and standard deviation across the 50 folds were subsequently collected.

#### 2.6.5. Model-Agnostic Explainability

After each fold, feature importance was extracted in a model-specific yet standardized fashion: absolute coefficients for logistic regression, native importance vectors for tree-based ensembles and CatBoost, and permutation importance (five repeats) for models lacking an intrinsic measure. Importances were averaged across folds to obtain a stable ranking that supported the interpretation of the predictors’ contribution. This step was included to identify which variables contributed most consistently to caregiver risk stratification across different modeling approaches. A complementary cross-model overview of predictor influence is provided in Supplementary Table S3, which reports the rank order of predictor importance across the included classifiers together with a summary index of mean normalized importance.

## 3. Results

A total of 113 patients and their respective caregivers were enrolled. The sample included 79 stroke survivors, 19 with PD, and 15 with AD, ranging from mild to moderate stages. The patient-caregiver relationship included 56 sons or daughters, 47 partners or cohabitants, and 10 parents in the entire cohort. Distributions within the pathology groups were 33/38/8 for stroke, 16/3/0 for PD, and 7/6/2 for AD, respectively.

Overall, patients’ MoCA score averaged 20.4 ± 6.5 (mean ± SD). The caregivers’ burden was expressed by the CBI, averaging 18.9 ± 15.9 (mean ± SD) points in the total score. Caregiver burnout risk was classified as absent or mild in 77 cases, moderate in 18 and severe in 18. The corresponding counts for stroke, PD, and AD were 46/17/16, 19/0/0, and 12/1/2, respectively.

After dichotomization, the moderate-to-severe class comprised 33 stroke caregivers, no PD caregivers, and three AD caregivers. A complete set of pathology-specific classification metrics was not estimated because the PD subgroup contained only one outcome class, whereas the AD subgroup included only three moderate-to-severe cases.

Detailed demographic and clinical characteristics of patients and caregivers are reported in Table 2.

Patient’s sex distribution differed by pathology ($\chi^{2}$ = 10.22, *p* = 0.006), whereas caregiver sex showed no association with pathology ($\chi^{2}$ = 0.34, *p* = 0.846). The caregiver–patient relationship profile also varied across pathology groups ($\chi^{2}$ = 11.62, *p* = 0.020). Caregiver burnout-risk levels differed by pathology as well ($\chi^{2}$ = 13.68, *p* = 0.008), with moderate/severe risk more frequent in stroke than in AD and not observed in the PD group.

### 3.1. Group Comparisons of Cognitive and Caregiver-Burden Measures

Patient cognitive performance and caregiver-burden scores were compared across the three pathology groups with non-parametric tests, since assumptions of normality or homogeneity of variance were violated for at least one group for every variable examined. The MoCA score differed significantly among groups (Kruskal-Wallis H = 15.6, *p* < 0.001). Post-hoc Dunn tests, corrected for false discovery rate, showed that patients with PD scored higher than those with stroke (*p* < 0.001) and AD (*p* = 0.008), whereas the stroke and ad groups did not differ (*p* = 0.894). For the CBI objective burden sub-scale, a global difference was also detected (H = 13.8, *p* = 0.001). Caregivers of stroke survivors reported greater objective burden than caregivers of PD (*p* = 0.009) and of persons with AD (*p* = 0.010), while the latter two groups were similar (*p* = 0.995). The CBI emotional sub-scale varied across groups (H = 8.2, *p* = 0.017); post-hoc analysis indicated higher emotional burden in AD caregivers compared with both stroke (*p* = 0.015) and PD caregivers (*p* = 0.031). No significant group differences emerged for the CBI developmental (*p* = 0.597), physical (*p* = 0.437), social (*p* = 0.232) or total scores (*p* = 0.132).

### 3.2. Correlation Analysis Between Predictors and Caregiver-Burden Measures

**Figure 1:**
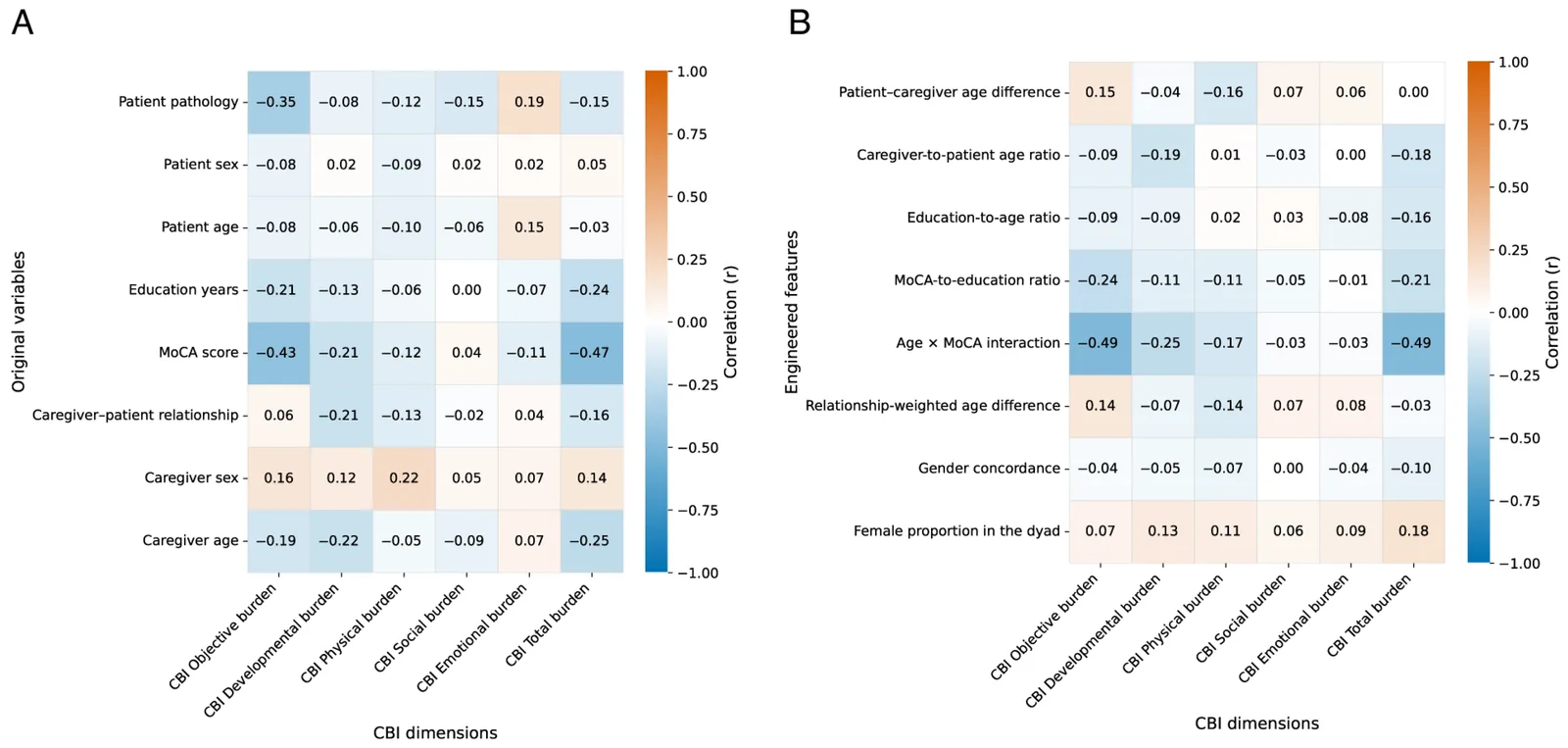
Correlations between predictors and Caregiver Burden Inventory (CBI) scores. Heatmaps report bivariate correlations between the (**A**) original (base) predictors and (**B**) engineered predictors and the Caregiver Burden Inventory (CBI) domains and total score. For each predictor–outcome pair, Pearson’s r was used when both variables met normality assumptions; otherwise Spearman’s rank correlation was used. Cell values show the correlation coefficient, with colour indicating direction and magnitude (blue = negative, red = positive; intensity proportional to | r |).

Figure 1 summarizes the correlation between the predictors and the CBI domain and total scores. Five predictor-outcome pairs met the dual criteria of statistical significance after false-discovery-rate correction (*p* < 0.05) and practical relevance (|r| ≥ 0.30). Patient MoCA alone was likewise inversely related to the CBI total (r = −0.47, *p* < 0.001) and Time Dependence (r = −0.43, *p* < 0.001) scores. Furthermore, patient pathology correlated modestly with the CBI Time Dependence domain (r = −0.35, *p* = 0.001), reflecting higher objective burden in stroke caregivers compared with those of PD (Figure 1A). Finally, the composite age × MoCA interaction showed a moderate inverse association with both the CBI Time Dependence domain (Spearman r = −0.49, *p* < 0.001) and the CBI total score (r = −0.49, *p* < 0.001), indicating that lower cognitive performance in older patients was linked to higher caregiver burden (Figure 1B).

### 3.3. Machine Learning Classification Results

Table 3 summarizes the performance obtained with each model.

**Figure 2:**
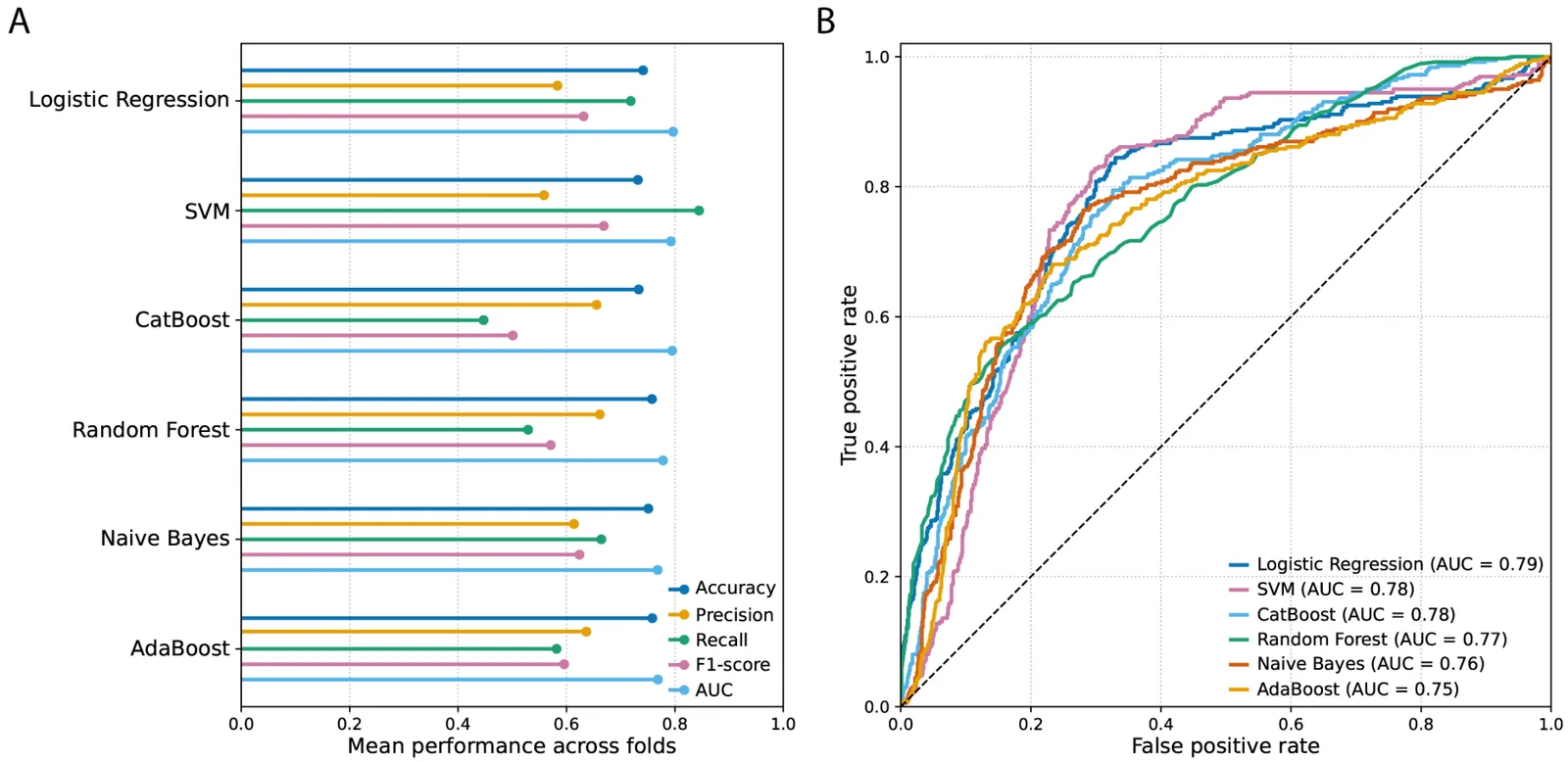
Cross-validated classification performance of the six models for caregiver burnout-risk stratification. (**A**) Mean performance across the 50 validation folds (5-fold stratified cross-validation repeated 10 times). Accuracy, precision, recall and F1-score refer to the positive class (moderate-to-severe burnout risk), and are shown on a 0–1 scale together with the mean area under the curve (AUC). (**B**) Receiver-operating characteristic (ROC) curves. Models are ordered by pooled AUC (highest to lowest), with pooled AUC values reported in the legend. Higher metric values indicate better classification performance. Recall corresponds to sensitivity for the moderate-to-severe class. In the ROC panel, the dashed diagonal represents chance-level discrimination (AUC = 0.50), whereas curves closer to the upper-left corner indicate better discrimination. SVM = support-vector machine.

Accuracy values ranged from 73.1% to 75.8% across models, with AdaBoost obtaining the highest mean accuracy (75.8% ± 8.6%). Precision peaked in Random Forest (66.2%), whereas the largest recall (84.5%) and the highest F1-score (66.9%) were achieved by the SVM with an RBF kernel. The greatest area under the receiver-operating-characteristic curve (AUC = 0.80) was observed in both Logistic Regression and CatBoost. An asterisk in Table 3 marks the best result within each metric column. Model performances are summarized in Figure 2.

### 3.4. Feature Importance

**Figure 3:**
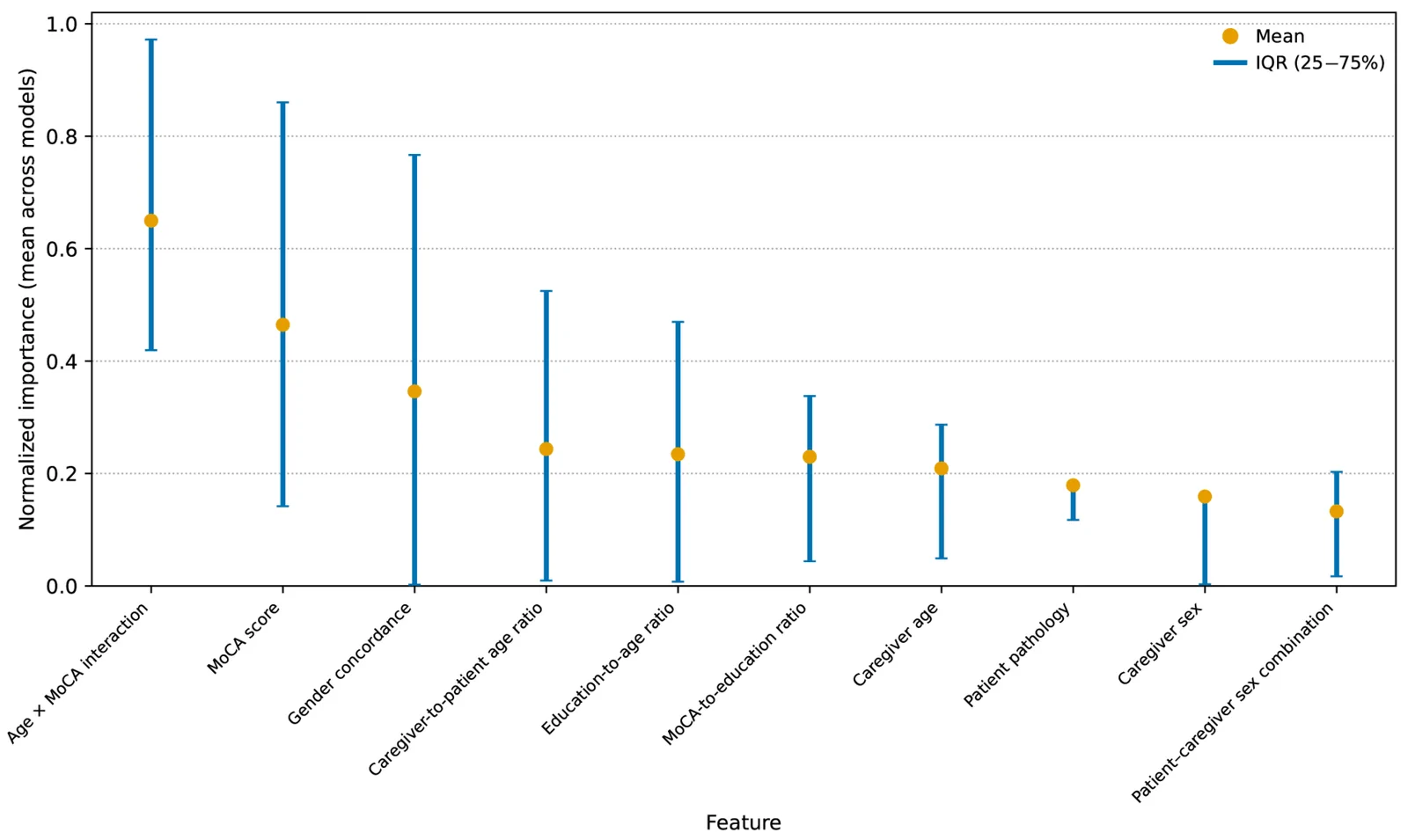
Global feature importance across models. Points indicate the mean feature importance averaged across the six classifiers, after normalizing importances within each model (maximum = 1). Vertical whiskers represent the interquartile range (IQR; 25th–75th percentile) across models. Features are ordered by decreasing mean normalized importance. Higher values indicate a greater relative contribution to the classification models. Wider interquartile ranges indicate greater variability in feature importance across classifiers. Feature importance reflects predictive contribution but does not indicate the direction of the association or imply causality.

Figure 3 reports the ten highest-ranked predictors, summarizing their mean normalized importance across the six classifiers (averaged over 50 cross-validation folds) and the corresponding interquartile range (25th–75th percentile) across models.

Across the six classifiers, the Age × MoCA interaction emerged as the most influential predictor overall, ranking first in both the SVM (RBF) and CatBoost models and remaining among the most heavily weighted inputs in AdaBoost, Random Forest, and Naïve Bayes. The MoCA score was the next most prominent contributor, ranking first in the Logistic Regression and Naïve Bayes models and second in CatBoost, supporting a central role of raw cognitive status in discriminating burnout-risk classes. Among the dyadic demographic descriptors, gender concordance (patient and caregiver share the same sex) showed high relevance primarily in the ensemble learners, where it was the leading feature in Random Forest and AdaBoost.

## 4. Discussion

The present study investigated determinants of caregiving burden across three major neurological conditions (Stroke, PD, and AD), in a single-center clinical sample.

Distinct cognitive profiles emerged across disease groups, with PD patients showing higher MoCA scores than stroke and AD groups. Disease-specific patterns of caregiving burden were identified, with stroke caregivers reporting greater time-dependent burden and AD caregivers experiencing greater emotional burden. Notably, the absence of between-group differences in total CBI scores indicates that global burden indices may obscure clinically meaningful domain-specific stressors. Cognitive impairment emerged as a central determinant of caregiving burden, particularly in interaction with advanced age. Finally, machine learning models further corroborate these findings, identifying cognitive performance, the age × cognition interaction, and patient–caregiver gender concordance as key predictors of moderate-to-severe burden risk.

Gender concordance (i.e., when patient and caregiver share the same sex) emerged as a relevant predictor based on feature importance in the machine learning models, suggesting that shared gender-related roles, expectations, or communication styles may influence caregiving dynamics and perceived burden. However, this finding should be interpreted cautiously and warrants further investigation.

These findings are consistent with previous studies indicating that residual disability after stroke is a major cause of objective burden, as daily assistance with motor and functional activities places a high burden on caregivers [7,25,26,27]. This interpretation is further supported by studies showing that post-stroke functional dependence, particularly in mobility and activities of daily living, is strongly associated with increased time commitment and role overload among caregivers [28,29]. Greater motor impairment and reduced autonomy have been identified as key predictors of caregiver strain in both the early and chronic phases following stroke [30,31], reinforcing the notion that stroke-related burden is predominantly objective and time-intensive in nature.

Similarly, the elevated emotional distress observed in caregivers of patients with AD highlighting the destabilizing impact of neuropsychiatric symptoms and behavioral changes on family members [11]. Some research has demonstrated that behavioral disturbances, progressive cognitive decline, and loss of relational reciprocity are major contributors to psychological distress, depressive symptoms, and emotional exhaustion in dementia caregivers [32,33].

Notably, neuropsychiatric symptoms such as agitation, apathy, and disinhibition often exert a stronger impact on caregiver emotional burden than functional impairment alone [34,35], suggesting that the affective dimension of caregiving strain is particularly salient in AD.

The lack of significant differences in total burden scores between groups suggests that global indices may underestimate specific stressors affecting caregivers in distinct conditions, supporting the need for a multidimensional assessment. As previously highlighted in caregiving research, composite burden measures may obscure clinically meaningful domain-specific variations [36,37], so it is important to distinguish between objective, emotional, physical, and relational components of caregiving strain when comparing different neurological populations.

The inverse associations between MoCA scores and caregiver burden reinforce the clinical relevance of patients’ cognitive status in shaping caregiving experiences. Beyond global cognitive decline, executive dysfunction and attentional deficits may be particularly burdensome, as they increase supervision demands, disrupt daily routines, and reduce patients’ ability to initiate or regulate behavior independently [34,38]. Cognitive impairment, particularly when associated with advanced age, appears to increase overall and time-dependent caregiving burden, likely due to increased supervision demands. The interaction between age and cognition may reflect cumulative vulnerability, whereby older patients with cognitive decline require more sustained monitoring and assistance, thereby amplifying caregiver time commitment and perceived responsibility. This finding is consistent with previous studies that have linked cognitive deficits to caregiver distress in neurological populations [39]. Meta-analytic evidence further indicates that cognitive impairment is among the strongest predictors of caregiver burden across dementia and mixed neurological samples [18,35], underscoring its central role in shaping dyadic stress processes.

The importance of gender concordance as a predictor adds a new perspective: when patient and caregiver are of the same sex, caregiving dynamics can intensify role expectations and emotional involvement, exacerbating caregiving burden.

From a sociocultural standpoint, gender concordance may activate normative role of scripts and expectations of caregiving reciprocity [40]. In same-sex dyads such as mother–daughter or husband–wife pairs, potentially increasing emotional identification and perceived obligation [41].

Similar patterns have been observed in oncology, where mother-daughter dyads have shown higher levels of stress [42,43], and in dementia care, where gender norms shape caregiving experiences [41].

These findings align with broader caregiving literature indicating that women, who are more likely to assume primary caregiving roles, report higher levels of emotional strain and depressive symptoms [44,45], suggesting that gender concordance may interact with established gender disparities in caregiving burden.

From a clinical perspective, these findings highlight the importance of early screening of caregivers at risk of high caregiver burden, as key predictors such as cognitive impairment (MoCA), its interaction with age, and dyadic characteristics may help identify vulnerable caregiver–patient pairs at an earlier stage.

Because these variables are routinely collected during standard neurorehabilitation assessments, they could be readily incorporated into existing multidisciplinary caregiver screening pathways without substantially increasing assessment burden. Routine assessment of caregiver strain, particularly in the early phases of disease management, may allow timely identification of vulnerable dyads before burden escalates into clinically significant distress or burnout [18]. Individualized interventions may include cognitive rehabilitation programs to mitigate patient-related risk factors, physical support services for caregivers of stroke survivors, and psychological support strategies for caregivers of individuals with AD. Importantly, improving patients’ cognitive and functional autonomy through targeted rehabilitation may indirectly reduce supervision demands and time-dependent burden, thereby exerting a secondary protective effect on caregiver well-being [46].

Furthermore, machine learning classifiers based on routinely collected clinical and dyadic variables may offer a complementary approach to caregiver risk stratification by capturing multivariable, non-linear, and interaction patterns [47]. Although the present findings suggest potential relevance for the identification of caregivers with clinically meaningful burden, the added value of model-generated predictions within routine clinical decision making remains to be established prospectively.

Recent work has shown that supervised Machine learning can generate interpretable, multivariable risk profiles for caregiver outcomes (including burden/burnout) across neurological cohorts, suggesting practical value for screening and triage when coupled with clinically meaningful explanations [48].

Notably, despite differences in their underlying assumptions and modeling strategies, the principal predictors identified in the present study—particularly the Age × MoCA interaction, MoCA score, and patient–caregiver gender concordance—consistently emerged among the most influential variables across the different classifiers. This convergence supports the robustness of these predictors, whereas differences in their relative ranking likely reflect the distinct abilities of the algorithms to capture non-linear relationships and feature interactions. Importantly, the reported classification performance refers to the pooled neurological sample and should not be interpreted as separate validation of the models within each pathology subgroup.

However, although the best-performing model in the present study reached classification accuracy up to 76%, such performance should be interpreted as moderate rather than definitive, reflecting the intrinsic complexity and contextual variability of caregiving dynamics. Therefore, the present findings should be regarded as preliminary and hypothesis-generating, providing a basis for further validation and prospective evaluation of their potential clinical application. External validation on independent cohorts and calibration analyses will be essential before clinical implementation, in order to ensure robustness, transportability, and real-world applicability of predictive models.

## 5. Strengths, Limitations, and Future Directions

The study has several notable strengths.

First, the direct comparison of three highly prevalent neurological conditions within a single, clinically characterized cohort minimizes methodological heterogeneity and allows for disease-specific contrasts under comparable assessment conditions.

Second, the combined use of traditional inferential statistics and machine learning approaches provides complementary analytical perspectives. One focused on hypothesis-driven group differences and the other on multivariable risk pattern detection. The identification of predictive factors spanning clinical, cognitive, and relational domains advances a multidimensional, dyadic conceptualization of caregiver burden and moves beyond disease-centered interpretations toward an integrated biopsychosocial framework.

Finally, the identification of interaction-based and dyadic patterns, such as the age × MoCA interaction and patient–caregiver gender concordance, represents an additional strength of the study, as it moves beyond traditional single-variable approaches and provides a more integrative understanding of caregiver burden.

However, several limitations should be considered when interpreting the findings.

First, the absence of differences across some caregiver burden subscales may reflect both sample heterogeneity and limited statistical power within specific domains.

Moreover, no formal a priori sample size estimation was performed, since the study was based on consecutive recruitment in a real-world clinical setting. The relatively limited sample size may have affected the stability and generalizability of the machine learning models and may increase the risk of overfitting; therefore, these findings should be interpreted with caution. In addition, the three pathology groups were unequally represented, with stroke dyads comprising most of the sample. Although this distribution reflected consecutive recruitment in the clinical setting, it may have reduced the statistical power and stability of comparisons involving the smaller PD and AD groups and may have given the stroke subgroup greater influence in the pooled machine learning analyses. Moreover, after outcome dichotomization, the PD subgroup contained no moderate-to-severe cases and the AD subgroup contained only three such cases. Reliable pathology-specific estimates of sensitivity and AUC could therefore not be obtained, and apparently favorable values for metrics such as accuracy or specificity would have been strongly influenced by the predominance of the mild class. Accordingly, the reported classification metrics apply to the pooled neurological sample and should not be interpreted as evidence of equivalent predictive performance within stroke, PD, and AD considered separately. The disease-specific findings, particularly those concerning PD and AD, should therefore be regarded as exploratory. Confirmation in larger cohorts with a more balanced representation of both pathologies and outcome classes is required.

Furthermore, dichotomizing the CBI score for the machine learning classification task may have reduced the information captured by the original continuous measure, although this approach was adopted to facilitate clinically meaningful caregiver risk stratification.

Second, the cross-sectional design precludes causal inferences and does not allow examination of the temporal evolution of caregiver burden. Without longitudinal follow-up, it remains unclear whether cognitive decline precedes increases in caregiver burden or whether dyadic stress processes develop dynamically over time. Furthermore, several psychosocial variables known to influence caregiver burden were not systematically assessed. Factors such as coping strategies, caregiver resilience and dedicated neuropsychological profiling, relationship type (e.g., spouse vs. offspring), duration and severity of disease, and socioeconomic resources may substantially moderate burden trajectories and were not included in the present models. Furthermore, no harmonized measure of physical disease severity or functional independence was available across all three neurological conditions. Disease-specific instruments, such as the National Institutes of Health Stroke Scale (NIHSS) for stroke, the Hoehn and Yahr scale for PD, and dementia severity scales, assess different constructs and could not be directly combined as equivalent raw predictors in the pooled analysis. Although the MoCA provided a common measure of cognitive functioning, it does not capture overall physical or clinical severity. Consequently, residual confounding related to motor impairment, disability, and dependence in activities of daily living cannot be excluded, particularly for the physical and time-dependence domains of caregiver burden. Future studies should collect common functional measures across diagnoses and, where sample sizes permit, incorporate disease-specific severity scores through stratified or appropriately standardized analyses. Moreover, the time since diagnosis was not systematically assessed and may differ across neurological conditions, potentially influencing disease severity and caregiver burden. In addition, the amount of time spent caregiving was not directly quantified (e.g., hours per day) and was only indirectly reflected by the time-dependence domain of the CBI. In particular, caregiver anxiety (both state and trait) was not directly assessed.

Although the CBI includes an emotional domain, it does not specifically capture anxiety as a distinct construct; therefore, its potential contribution to caregiver burden could not be disentangled in the present analysis. The absence of these variables may have limited the explanatory capacity of both traditional and machine learning analyses.

Furthermore, although the caregiver-patient relationship is likely to influence burden patterns, formal within-pathology comparisons by relationship type were not performed because subgroup sizes were unbalanced and in some cases too small to support reliable inference. Larger studies specifically powered for relationship-stratified analyses will be needed to clarify these effects across neurological conditions.

Although the present models relied on standardized demographic, clinical, and neuropsychological measures, future predictive approaches may benefit from integrating multimodal biomarkers to provide a more comprehensive characterization of patient status and disease progression. In particular, objective neurophysiological measures, such as electroencephalography (EEG), together with advanced machine learning and deep learning techniques, may complement conventional clinical assessments and improve caregiver risk stratification. Recent work has highlighted the potential of EEG-based deep learning approaches for monitoring neurological conditions, suggesting promising avenues for future predictive models [49,50].

Finally, the models were developed and evaluated exclusively within a single-center cohort, and no independent external dataset was available for validation. Although repeated stratified cross-validation provided an internal estimate of model performance across multiple data partitions, it cannot determine whether the observed accuracy and discrimination would be maintained in populations recruited under different clinical, demographic, or organizational conditions. The reported performance metrics may therefore be influenced by center-specific sampling characteristics, referral patterns, and assessment procedures. Validation in larger, independent, and preferably multicenter cohorts, together with assessment of calibration and subgroup-specific performance, will be required to establish robustness, transportability, and potential clinical utility. Accordingly, the predictive performance observed in this study should be considered preliminary and hypothesis-generating. Although the proposed models provide an initial proof of concept for caregiver burden risk stratification, their generalizability and clinical utility require confirmation through external validation in larger, independent cohorts before routine clinical implementation.

Beyond external validation, future clinical implementation of machine learning-based caregiver risk stratification will require careful consideration of data governance, patient privacy, cybersecurity, and regulatory compliance. Because the present models were developed and internally validated using retrospectively analyzed clinical data collected within an ethically approved observational protocol, issues related to real-time deployment, secure integration into hospital information systems, continuous model monitoring, and compliance with applicable data protection regulations (e.g., General Data Protection Regulation) were beyond the scope of the present study. Future translational research should specifically address these aspects before routine clinical implementation.

Furthermore, the clinical utility of the proposed models was not directly evaluated in the present study. Future prospective investigations will therefore be needed to determine whether model-based risk estimates provide meaningful added value alongside routine clinical assessment and can effectively support caregiver screening, referral, or intervention planning.

Future research should adopt longitudinal models to monitor caregiver burden throughout disease progression and integrate psychosocial and biological markers to refine predictive models. Prospective, dyadic designs would allow examination of temporal dynamics and reciprocal influences between patient deterioration and caregiver stress trajectories. Scaling these approaches toward routine use will require evaluation on larger, more diverse datasets to better characterize generalizability across clinical contexts and caregiver–patient profiles.

Attention should be given to the inclusion of socio-demographically diverse populations to enhance model transportability and reduce potential algorithmic bias.

In addition to internal cross-validation, models should be assessed through external validation on independent cohorts, with emphasis on calibration, stability of performance across relevant subgroups (e.g., pathology, age, sex, relationship type), and clinical utility. Beyond discrimination accuracy, future studies should evaluate clinical interpretability and decision-support value to ensure meaningful integration into real-world screening pathways.

## 6. Conclusions

In conclusion, our study highlights both shared and disease-specific determinants of caregiver burden in stroke, PD, and AD. Cognitive impairment, patients’ age, and gender concordance between patient and caregiver emerged as key risk factors. These findings support a multidimensional, dyadic framework in which cognitive impairment and related clinical characteristics, along with relational dynamics, jointly shape caregiving strain. Rather than viewing caregiver burden as a uniform construct, our results underscore the importance of domain-specific assessment and tailored intervention strategies.

Future research should further investigate the potential of advanced analytical approaches for caregiver risk stratification. Before these models can be integrated into routine clinical practice, future studies should externally validate these models and address data governance, privacy, regulatory, and implementation requirements.

## Figures and Tables

**Table 1:** Summary of factors associated with caregiver burden in neurological disorders.

Category	Factors
**Patient-related factors **	Clinical status; neuropsychiatric symptoms; premorbid personality traits; cognitive impairment; behavioral disturbances; physical disability.
**Disease-specific features**	PD: motor fluctuations, non-motor symptoms (e.g., depression, apathy); AD: cognitive decline, behavioral disturbances; Stroke: residual physical disability and cognitive impairment.
**Caregiver-related factors**	Sociodemographic characteristics; gender-related influences; adherence to gender role stereotypes.
**Psychosocial factors**	Lack of social support; presence of anxiety or depression.
**Dyadic factors**	Relationship between caregiver and patient (e.g., spouse, child), with differences in burden, identity shifts, and social isolation.

Legend: Summary of the main factors associated with caregiver burden in neurological disorders, grouped into broad conceptual domains based on the literature discussed in the Introduction. PD = Parkinson’s disease; AD = Alzheimer’s disease.

**Table 2:** Demographic and clinical characteristics of the study sample, stratified by patients’ pathology.

Pathology	Total	Stroke	PD	AD
***Patients ***	***n = 113***	***n = 79***	***n = 19***	***n = 15***
Age, years (mean ± SD)	61.2 ± 13.3	59.4 ± 14.6	62.8 ± 6.0	68.9 ± 9.6
Male, *n* (%)	65 (57.5)	49 (62.0)	13 (68.4)	3 (20.0)
Female, *n* (%)	48 (42.5)	30 (38.0)	6 (31.6)	12 (80.0)
Education, years (mean ± SD)	10.64 ± 4.03	10.46 ± 4.04	12.32 ± 3.80	9.47 ± 3.87
MoCA (mean ± SD)	20.4 ± 6.5	19.2 ± 7.0	25.4 ± 2.5	20.1 ± 4.4
MoCA range (min–max)	5–30	5–30	20–30	11–25
***Caregivers***	***n = 113***	***n = 79***	***n = 19***	***n = 15***
Age, years (mean ± SD)	54.7 ± 12.1	52.7 ± 12.3	59.5 ± 9.9	59.1 ± 11.7
Male, *n* (%)	39 (34.5)	26 (32.9)	7 (36.8)	6 (40.0)
Female, *n* (%)	74 (65.5)	53 (67.1)	12 (63.2)	9 (60.0)
Familiarity–Son/Daughter, *n* (%)	56 (49.6)	33 (41.8)	16 (84.2)	7 (46.7)
Familiarity–Spouse/Partner, *n* (%)	47 (41.6)	38 (48.1)	3 (15.8)	6 (40.0)
Familiarity–Parent, *n* (%)	10 (8.8)	8 (10.1)	0 (0.0)	2 (13.3)
CBI–Time Dependence (mean ± SD)	8.7 ± 6.6	10.2 ± 7.2	5.1 ± 2.2	5.3 ± 3.4
CBI–Developmental (mean ± SD)	4.7 ± 4.8	4.9 ± 5.0	4.2 ± 3.5	4.0 ± 5.2
CBI–Physical (mean ± SD)	5.3 ± 4.6	5.7 ± 4.9	4.6 ± 3.4	3.9 ± 3.9
CBI–Social (mean ± SD)	2.8 ± 3.6	3.0 ± 3.5	2.3 ± 2.5	2.6 ± 5.0
CBI–Emotional (mean ± SD)	1.5 ± 2.5	1.3 ± 2.2	1.0 ± 1.3	3.3 ± 3.9
CBI–Total (mean ± SD)	18.9 ± 15.9	20.6 ± 16.2	11.9 ± 8.5	19.1 ± 19.9

Legend: Demographic and clinical characteristics of the study cohort shown for the total sample and stratified by pathology (stroke, PD = Parkinson’s disease, AD =Alzheimer’s Disease). Continuous variables are reported as mean ± standard deviation (SD); categorical variables are expressed as number (percentage). Familiarity indicates the caregiver’s relationship with the patient. MoCA = Montreal Cognitive Assessment. CBI = Caregiver Burden Inventory, reported for each sub-scale and as a total score (higher values denote greater burden).

**Table 3:** Classification performance of the included machine learning models.

Model	Accuracy % (Mean ± SD)	Precision %	Recall %	F1-Score %	AUC
AdaBoost	75.83 ± 8.61 *	63.67	58.18	59.58	0.77
Random Forest	75.77 ± 7.76	66.15 *	52.93	57.10	0.78
Logistic Regression	74.13 ± 7.91	58.33	71.86	63.16	0.80 *
SVM (RBF)	73.18 ± 7.43	55.87	84.46 *	66.88 *	0.79
Naïve Bayes	75.11 ± 8.21	61.39	66.43	62.40	0.77
CatBoost	73.33 ± 7.50	65.56	44.71	50.10	0.80 *

Mean performance across 50 cross-validation folds for each machine learning classifier. Accuracy is reported as mean ± SD; precision, recall and F1-score are expressed as percentages. SD = standard deviation; AUC = area under the receiver-operating-characteristic curve; SVM = support-vector machine; RBF = radial-basis function. An asterisk (*) marks the best value achieved in each column.

## Data Availability

The data presented in this study are not publicly available due to privacy or ethical restrictions but are available on request from the corresponding author.

## Supplementary Materials

The following supporting information can be downloaded at: https://www.mdpi.com/article/10.3390/medsci14040428/s1, Table S1: Full list of variables used in the machine learning framework; Table S2: Final parameter sets for the included machine learning models; Table S3: Cross-model overview of predictor influence and mean normalized importance.

## Abbreviations

The following abbreviations are used in this manuscript:

AD	Alzheimer’s disease
ANOVA	Analysis of variance
AUC	Area under the receiver-operating-characteristic curve
CBI	Caregiver Burden Inventory
EEG	Electroencephalography
IQR	Interquartile range
MoCA	Montreal Cognitive Assessment
NIHSS	National Institutes of Health Stroke Scale
PD	Parkinson’s disease
RBF	Radial-basis function
ROC	Receiver-operating characteristic
SD	Standard deviation
SVM	Support-vector machine
