# Supplementary material for: Identifying Risk Factors for Caregiver Burden in Neurological Disorders Using Machine Learning
Source: Med Sci (Basel). 2026 Jul 25;14(4):428. doi: 10.3390/medsci14040428 (PMC13515725; doi:10.3390/medsci14040428)
Supplement: Supplementary file 1 [file medsci-14-00428-s001.zip › medsci-4444077-supplementary.pdf]

Supplementary Materials

Identifying Risk Factors for Caregiver Burden in Neurological Disorders using Machine Learning

Maria Grazia Maggio, Augusto Ielo, Rosaria De Luca, Francesco Corallo, Angela Marra, Davide Cardile, Amelia Rizzo, Angelo Quartarone and Rocco Salvatore Calabrò

Supplementary Tables

Table S1: Candidate predictors used for machine learning models, with composite features and their definitions.

**Variable name**	**Type**	**Definition**
Patient pathology	Base	Patient pathology group
Patient sex	Base	Patient sex, coded 1 = male and 2 = female
Patient age	Base	Patient age in years
Education years	Base	Years of formal education
MoCA score	Base	Montreal Cognitive Assessment total score
Caregiver–patient relationship	Base	Relationship, coded 1 = son/daughter, 2 = spouse/partner, 3 = parent
Caregiver sex	Base	Caregiver sex, coded 1 = male and 2 = female
Caregiver age	Base	Caregiver age in years
Patient–caregiver age difference	Composite	Absolute difference between patient and caregiver age: |patient age − caregiver age|
Caregiver-to-patient age ratio	Composite	Ratio of caregiver to patient age: caregiver age / patient age
Education-to-age ratio	Composite	Education normalized by age: education years / patient age
MoCA-to-education ratio	Composite	MoCA normalized by education: MoCA score / education years
Age × MoCA interaction	Composite	Product of age and cognition: patient age × MoCA score
Pathology-by-age group	Composite	Combined pathology and age group: patient pathology + "_" + age group
Relationship-weighted age difference	Composite	Relationship-weighted age difference: relationship code × patient–caregiver age difference
Gender concordance	Composite	Indicator equal to 1 when patient and caregiver share the same sex, otherwise 0
Patient–caregiver sex combination	Composite	Combined patient and caregiver sex: patient sex + "_" + caregiver sex
Sex-by-relationship combination	Composite	Combined sex combination and relationship: patient–caregiver sex combination + "_" + relationship code
Female proportion in the dyad	Composite	Proportion of females in the dyad: [(patient sex = female) + (caregiver sex = female)] / 2 (values 0, 0.5, 1)

Legend: Base (original) variables were directly available from the clinical dataset. Composite (engineered) variables were derived from original variables to capture clinically interpretable relationships, such as differences, ratios, interactions, or dyadic indicators.

Table S2: Hyper-parameters used for each machine learning model.

**Model**	**Hyper-parameters**
Logistic Regression	C = 0.01; solver = liblinear; class_weight = balanced;
Random Forest	n_estimators = 200; max_depth = None; class_weight = balanced;
AdaBoost	n_estimators = 200; learning_rate = 0.1;
SVM	kernel = rbf; C = 1; gamma = scale; probability = True; class_weight = balanced;
Naïve Bayes	default scikit-learn settings (Gaussian NB)
CatBoost	depth = 3; learning_rate = 0.01; iterations = 100;

Legend: Hyper-parameters correspond to the final settings selected during an initial, fully cross-validated grid search on the development data, and then held fixed for all subsequent repeated 5-fold stratified cross-validation runs reported in this study. class_weight = balanced indicates automated inverse-frequency weighting of classes when supported. For Gaussian naïve Bayes, the default implementation was used and no additional hyper-parameters were set in the script.

Table S3: Rank order of predictor importance across the included machine learning classifiers. Within each model, predictors were ranked from highest to lowest importance according to the model-specific importance metric (1 = highest importance). Dashes indicate null or negligible importance. For models using permutation importance, negative values were truncated to zero before ranking. Mean normalized importance is reported only as a cross-model summary index for ordering predictors and should not be interpreted as a standardized regression coefficient or significance estimate.

**Predictor**	**LR**	**RF**	**Ada**	**SVM**	**NB**	**Cat**	**Mean norm. imp.**
age_moca_interaction	14	5	3	1	2	1	0.649
patient_moca	1	10	7	–	1	2	0.465
same_gender	18	1	1	–	–	17	0.346
age_ratio	5	2	4	–	–	9	0.265
edu_age_ratio	7	4	2	–	–	13	0.246
moca_edu_ratio	4	8	13	–	3	4	0.229
caregiver_age	3	7	11	–	5	5	0.209
patient_pathology	6	11	6	–	7	3	0.179
caregiver_sex	2	13	8	–	–	16	0.173
gender_combo	9	9	12	–	11	10	0.133
familiarity_age_diff	15	3	5	2	–	12	0.125
age_diff	8	12	13	–	10	7	0.122
patient_education_years	10	17	17	–	4	8	0.114
pathology_age_group	19	6	15	–	8	6	0.090
female_proportion	13	15	10	–	6	15	0.088
caregiver_familiarity	11	14	9	–	9	11	0.082
gender_familiarity	12	18	–	–	–	–	0.039
patient_age	16	15	16	–	–	14	0.032
patient_sex	17	19	–	–	–	18	0.017

Legend: LR = Logistic Regression; RF = Random Forest; Ada = AdaBoost; SVM = support-vector machine with radial-basis-function kernel; NB = Naïve Bayes; Cat = CatBoost. Mean norm. imp. = mean normalized importance across models.
